# Psychedelic-Assisted Psychotherapy: A Paradigm Shift in Psychiatric Research and Development

**DOI:** 10.3389/fphar.2018.00733

**Published:** 2018-07-05

**Authors:** Eduardo Ekman Schenberg

**Affiliations:** Phaneros, São Paulo, Brazil

**Keywords:** psychedelic-assisted psychotherapy, LSD, MDMA, ibogaine, psilocybin, ketamine, explanation in neuroscience, states of consciousness

## Abstract

Mental disorders are rising while development of novel psychiatric medications is declining. This stall in innovation has also been linked with intense debates on the current diagnostics and explanations for mental disorders, together constituting a paradigmatic crisis. A radical innovation is psychedelic-assisted psychotherapy (PAP): professionally supervised use of ketamine, MDMA, psilocybin, LSD and ibogaine as part of elaborated psychotherapy programs. Clinical results so far have shown safety and efficacy, even for “treatment resistant” conditions, and thus deserve increasing attention from medical, psychological and psychiatric professionals. But more than novel treatments, the PAP model also has important consequences for the diagnostics and explanation axis of the psychiatric crisis, challenging the discrete nosological entities and advancing novel explanations for mental disorders and their treatment, in a model considerate of social and cultural factors, including adversities, trauma, and the therapeutic potential of some non-ordinary states of consciousness.

## The current psychiatric crisis

Mental disorders increasingly contribute to the global burden of disease, with huge socio-economic costs (Catalá-López et al., 2013; Whiteford et al., 2013). However, research and development in psychopharmacology—psychiatry's primary mode of intervention—came to a halt in 2010 (Miller, 2010; Hyman, 2013). Approval of new molecular entities for psychiatric conditions by the US Food and Drug Administration (FDA) fell from 13 in 1996 to one in 2016, with 49 approved between 1996 and 2006 and 22 from 2007 to 2016^1^ In pharmacology conferences in the period, just about 5% of presentations were dedicated to human studies involving drugs with novel mechanisms of action (van Gerven and Cohen, 2011). These occurrences are part of a complex picture clearly dissected as a triple crisis in psychiatry: of therapeutics, diagnostics and explanation (Rose, 2016).

Problems surrounding psychiatric diagnosis also surfaced in 2010, when the UK Medical Research Council published a strategy for mental health and wellbeing (Sahakian et al., 2010) and the US National Institute for Mental Health (NIMH) launched its Research Domain Criterion (RDoC). It proposed five domains based on specific neural systems that can be impaired in mental illness, a radical departure from the hundreds of discrete conceptual disorders of the much older Diagnostic and Statistical Manual (DSM) (Casey et al., 2013; Insel, 2014; Kraemer, 2015). Thus, the RDoC advanced a multidimensional approach to diagnosing mental disorders in a continuous spectra (Adam, 2013). At around the same time, a network psychopathology perspective was conceptualized and empirically assessed with statistical models for psychometrics based on thousands of patient reports' and hundreds of symptoms (Fried et al., 2017).

The treatment and diagnostic axes of the crisis are connected by the explanatory domain: despite huge investment in neuroscience as the ultimate source for understanding mental illness, both classification and diagnosis (Stephan et al., 2016a) as well as knowledge about pathogenesis and etiology still faces many challenges (Stephan et al., 2016b). The explanatory debate about mental disorders is summarized by the contrasting declarations that “mental disorders are brain disorders” (Deacon, 2013; Insel and Cuthbert, 2015) or that psychiatry runs the risk of “losing the psyche” (Parnas, 2014).

## Clinical developments with psychedelics

Synthetic substances like Lysergic Acid Diethylamide (LSD), 3,4-MethylenodioxyMetamphetamine (MDMA), 2-(2-Chlorophenyl)-2-(methylamino) cyclohexanone (ketamine) and naturally occurring alkaloids including 4-phosphoriloxy-N,N-dimethyltryptamine (psilocybin, present in hundreds of Psilocybe mushroom species) and 12-Methoxyibogamine (ibogaine, from Tabernanthe iboga) have been used in a series of studies (Passie et al., 2008; Brown, 2013; Tylš et al., 2014; Mithoefer et al., 2016; Nichols, 2016; for reviews see Winkelman, 2014; Dos Santos et al., 2016; Johnson and Griffiths, 2017; Nichols et al., 2017) as well as Phase 2 clinical trials (Table 1). These substances are orally active but have different mechanisms of action. LSD and psilocybin effects' critically depend on 5-HT_2A_ agonism, MDMA inhibits monoamine transporters, especially for serotonin, while ketamine is an NMDA antagonist and ibogaine non-specifically binds to many receptors.

**Table 1:** Registered Phase 2 clinical trials using psychedelics with psychiatric patients^*^.

**Substance**	**Diagnosis**	**Number of trials (ongoing/complete)**	**Number of patients (planned/complete)**	**Masking**	**Controls**	**Effect size (min/max)^°^**	**Published references**
**Ketamine** *Route of administration* (oral, intranasal, i.v.^$^) *Dose Range* (0.5–1.0 mg/kg oral, 0.2–0.5 mg/kg intranasal, 0.1–1.0 mg/kg i.v.) *Number of drug sessions* (1–12)	Depression^#^	42/13	3,309/504	Open label, single-blind, double blind	Placebo, lithium, saline, diphenhydramine, nitroprusside, midazolam, minocyclin, ECT	0.99–1.67	Fond et al., 2014; Coyle and Laws, 2015; Lee et al., 2015; McGirr et al., 2015; Parsaik et al., 2015; Romeo et al., 2015; Wan et al., 2015; Kishimoto et al., 2016; Xu et al., 2016
	OCD	8/4	171/35	Open label, double-blind	Placebo, saline, midazolam	0.8	Bloch et al., 2012; Rodriguez et al., 2013, 2016
	PTSD	5/1	318/41	Double-blind	Placebo, midazolam	NA	Feder et al., 2014
	Suicide^+^	5/1	718/12	Open label, double-blind	Saline, midazolam	0.67–0.84	Ballard et al., 2014; Price et al., 2014; Murrough et al., 2015
	Alcohol use disorder	3/0	221/0	Open label, double-blind	Placebo, midazolam	–	–
	Cocaine use disorder	2/2	68/8	Double-blind	Lorazepam	NA	Dakwar et al., 2014, 2016
	Subtotal	68/21	4,717/588^***^	–	–	–	–
**MDMA** *Route of administration* (oral) *Dose Range* (62.5–187.5 mg) *Number of drug sessions* (2–3)	PTSD	12/6	152/108	Open label, double-blind	Lactose, 25 mg MDMA, 30 mg MDMA	1.17–1.24	Bouso et al., 2008; Mithoefer et al., 2011, 2013, 2018; Oehen et al., 2013; Yazar-Klosinski and Mithoefer, 2017
	Social anxiety in autistic adults	1/0	12/0^§^	Double-blind	Inactive placebo	–	–
	Existential anxiety	3/0	18/0	Double-blind	Inactive placebo, 25 mg MDMA	–	–
	Alcohol use disorder	1/0	20/0	Open-label	Placebo	–	–
	Subtotal	17/6	202/120	–	–	–	–
**Psilocybin** *Route of administration* (oral) *Dose range* (10–40 mg) *Number of drug sessions* (1–3)	Depression	2/1	36/12	Open label, double blind	Diphenhydramine	2.0–3.1	Carhart-Harris et al., 2016, 2017c
	Existential anxiety	2/2^**^	80/80	Double-blind	Placebo, 4 mg psilocybin	0.82–1.63	Grob et al., 2011; Griffiths et al., 2016; Ross et al., 2016
	Alcohol dependence	2/1^$$^	190/10	Open-label, double blind	Diphenhydramine	1.19–1.39	Bogenschutz et al., 2015
	Cocaine related disorders	1/0	40/0	Double-blind	Diphenhydramine	–	–
	Cigarette dependence	1/1	15/15	Open-label	Transdermal nicotine patch	NA	Johnson et al., 2014, 2017b; Garcia-Romeu et al., 2015
	Subtotal	8/5	361/117	–	–	–	–
**LSD** *Route of administration* (oral) *Dose range* (200 μm) *Number of drug sessions* (2)	Existential anxiety	2/1	52/12	Double-blind	Mannitol, 20 mg LSD	1.1/1.2	Gasser et al., 2014
Total	–	95/34	5,332/837	–	–	–	–

* *Data obtained from clinicaltrials.gov and clinicaltrialsregister.eu, last access July, 21, 2017*.

# *Including registers for depression; major depression; depressive disorder; major depressive disorder; treatment resistant depression; bipolar depression; cancer depression; depression, suicide*.

+ *Including registers for Depression, suicide; MDD, suicide; BD, suicide; and suicidal ideas*.

** *Status at clinical trials.gov as ongoing, but both have already been published (Griffiths et al., 2016; Ross et al., 2016)*.

$$ *Not registered as completed, but published (Bogenschutz et al., 2015)*.

*** *There were 12 completed patients overlapping suicide and depression, 65 planned patients overlapping depression with alcohol use disorder and 16 planned patients overlapping depression with suicide. Each only counted once in the respective subtotals*.

$ *Generally infused over 40 min. °Reported as Standardized Mean Differences (Conhen's d), except psilocybin for depression, reported as Hedge's g. NA, Not Available at the published paper(s)*.

The most studied is ketamine, which in higher doses is an anesthetic in use for decades. In lower dosages it temporarily modify consciousness including changes in mood and cognition (Mion, 2017). It is the experimental intervention in almost 70 Phase 2 trials for psychiatric disorders and two Phase 3 trials for depression. Protocols involve single or repeated administrations in different doses, routes of delivery and research designs. Most are for depressive disorders, but is also studied for Obsessive-Compulsive Disorder (OCD), Post-Traumatic Stress Disorder (PTSD), suicide, alcohol, and cocaine use disorders (Table 1). Nine meta-analysis from depression trials (Fond et al., 2014; Coyle and Laws, 2015; Lee et al., 2015; McGirr et al., 2015; Parsaik et al., 2015; Romeo et al., 2015; Wan et al., 2015; Kishimoto et al., 2016; Xu et al., 2016) shows low frequency of serious adverse events in the short term (but see Short et al., 2017 for long-term reporting bias), with short-term positive outcomes for a significant proportion of patients.

MDMA is investigated in 17 Phase 2 trials (Table 1) and was designated a breakthrough therapy for PTSD by the FDA, a status that can expedite approval (Kupferschmidt, 2017). Also studied for social anxiety in autistic adults, existential anxiety and alcohol use disorder (Table 1), MDMA is commonly confused with the street drug “*ecstasy*” (also known as “*molly”*). However, these illegal products frequently do not contain MDMA, only adulterants (Vogels et al., 2009; Wood et al., 2011; Togni et al., 2015; Saleemi et al., 2017; Vrolijk et al., 2017). This loose terminology creates unfortunate confusion about MDMA's safety (Amoroso, 2016). In research with healthy volunteers, occurrences of hypertension, tachycardia and hyperthermia are below 1/3 of cases, not leading to serious adverse events (Vizeli and Liechti, 2017). In clinical populations, serious adverse events were very rare, with only one brief and self-limiting case of increased ventricular extrasystoles in more than 1,260 sessions (MAPS, 2017). Therapeutic results obtained with severe, treatment-resistant PTSD patients in Phase 2 studies were considered “spectacular” (Frood, 2012), with approximately 70% or more of participants no longer qualifying for the diagnosis after 12 months, while the remainder third had less intense symptoms. Furthermore, the improvements lasted up to 4 years, mostly without additional treatments and without inducing drug abuse or dependence (Mithoefer et al., 2013; Yazar-Klosinski and Mithoefer, 2017). An independent preliminary meta-analysis found MDMA-assisted psychotherapy was superior to prolonged exposure when evaluated by clinician-observed outcomes, by patient self-report outcomes and also by drop-outs (Amoroso and Workman, 2016).

Psilocybin is the third most studied psychedelic substance for clinical applications. It has a very high safety ratio (Gable, 2004; Tylš et al., 2014) and very low risk profile even in unsupervised settings (Nutt et al., 2010; van Amsterdam et al., 2011;)^2^ It's orally administered in eight trials for major depression, cigarettes, alcohol, and cocaine use disorders and existential anxiety in life-threatening diseases, mostly cancer. Despite moderately increasing blood pressure (Griffiths et al., 2011) and inducing transient headaches (Johnson et al., 2012), it has been safely administered to more than a 100 volunteers in neuroscientific research (Studerus et al., 2011) and another 100 in clinical studies with notable results (Table 1).

LSD, the most potent psychedelic currently administered in clinical trials, has very slow dissociation kinetics at the human 5-HT_2A_ receptor and thus long lasting effects (Wacker et al., 2017). It has a very high safety ratio (Gable, 2004; Passie et al., 2008) and is not associated with major health impairments after unsupervised use (Krebs and Johansen, 2013; Hendricks et al., 2014, 2015; Johansen and Krebs, 2015). It is the active substance in just two recent Phase 2 trials for existential anxiety in the terminally ill (Table 1). This paucity is perhaps due to stigma surrounding large-scale recreational use since the 1960's, with considerable political implications (Dyck, 2005; Nutt et al., 2013; Smith et al., 2014). However, before political turmoil, more than a 1,000 studies including 40,000 patients were done (Grinspoon, 1981), mostly showing positive potentials (Abraham et al., 1996). LSD was thus the prototypical substance in the development of radically new forms of psychotherapy, including psychedelic-assisted psychotherapy (Pahnke et al., 1970; Grof, 1971, 2008) and another approach based on repeated low doses (10 to 50 μg) to potentiate psychoanalysis, known as psycholytic psychoherapy (Majić et al., 2015). Despite the paucity of recent trials, a recent meta-analysis with rigorous research from 60 years ago confirmed LSD also has important potential for alcohol use disorders (Krebs and Johansen, 2012).

Finally, ibogaine is the less advanced psychedelic in the development pipeline, with no interventional clinical trials executed or registered since the National Institute on Drug Abuse (NIDA) cancelled efforts to develop this compound to treat opioid addiction in the 1990's (Alper, 2001). And indeed there are important safety concerns, given ibogaine can prolong QT interval (Koenig and Hilber, 2015), potentially evolving to fatal cardiac arrhythmias (Koenig et al., 2014). This critically differentiates ibogaine's safety profile from other psychedelics. However, given the seriousness of drug addiction and the difficulty to treat these patients, observational and retrospective studies for opioid (Brown and Alper, 2017; Noller et al., 2017) and psychostimulant addiction (Schenberg et al., 2014, 2016, 2017) reporting considerable success suggests Phase 2 trials focusing on cardiac safety should be performed. Given ibogaine is unscheduled in many countries and currently used as an alternative treatment with an unfortunate series of fatalities (Alper et al., 2012), financial support is needed.

## Psychedelic-assisted psychotherapy (PAP)

Safeguarded important differences regarding safety and mechanisms of action, the grouping of these substances in a prototypical PAP model has important practical and theoretical implications. The main feature is the therapeutic use of a potent psychoactive substance (currently most are scheduled compounds) in very few sessions. These are generally accompanied by drug-free sessions before and/or after drug sessions, usually called preparatory and integrative psychotherapy, respectively. With ketamine positive results were obtained with one to 12 administrations, with MDMA just three and with psilocybin and LSD only two, while ibogaine may be effective after a single administration. During drug effects, patients are continuously monitored and supported by trained mental health professionals following available guidelines (Johnson et al., 2008)^3^. Generally patients listen to instrumental evocative music (Pahnke et al., 1970; Bonny and Pahnke, 1972; Kaelen et al., 2015; Barrett et al., 2017; Richards, 2017) and are encouraged to stay introspective (with eyeshades) and open to feelings, attentive to thoughts and memories, being free to engage in psychotherapy at any time (Grof, 2008). Frequency and type of psychotherapeutic interventions varied from a minimum in ketamine studies, sometimes including only music during drug effects, to a more intensive protocol with MDMA including 12 non-drug sessions, which follow a detailed manual based on non-directive transpersonal psychology^3^ (recently, MDMA has also been tested with cognitive behavioral conjoint therapy). Between these two ends of the spectrum are psilocybin, LSD and ibogaine studies, which used a variety of interventions. Psilocybin studies used psychological support comprised of non-directive preparation, support and integration in few non-drug sessions. LSD included three post-drug integrative sessions. Ibogaine, used in different clinics for drug dependence, included a series of more or less standardized psychotherapies for addiction, pre- and post-drug, like 12-steps, individual and group counseling, among others. Increasing focus on types and frequency of psychotherapeutic interventions can arguably help improve outcomes, as exemplified by older ketamine studies with existentially oriented psychotherapy for drug addiction (e.g., Krupitsky and Grinenko, 1997; Krupitsky et al., 2007) and as recently tested with cognitive behavioral therapy for relapse prevention after ketamine for depression (Wilkinson et al., 2017). As results from most trials reliably show, PAP can be more effective and faster than current treatments, even for patients considered “treatment resistant.” And these outcomes were not only statistically significant but had large effect sizes, which is encouraging for Phase 3 trials.

**Figure 1:**
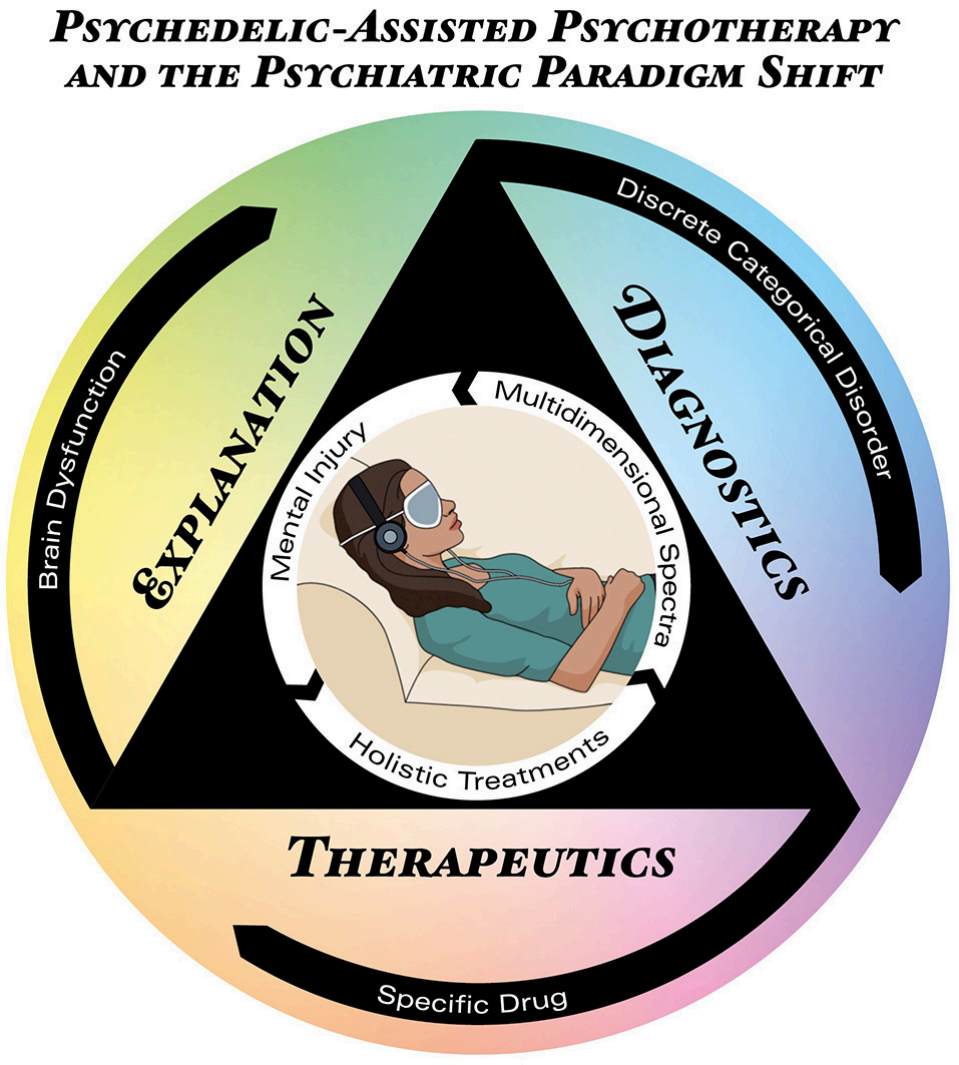
Psychedelic-Assisted Psychotherapy (PAP) mapped onto the triple-axis psychiatric crisis. The icon in the center represents the PAP model, located inside the triangle projecting the three axes of the current psychiatric crisis: therapeutics **(bottom)**, diagnosis **(right)**, and explanation **(left)**. The outermost black circle represents the main conceptual formulation for each axis in current psychiatric theory, i.e., brain dysfunctions diagnosed as discrete categorical disorders treated with specific drugs. The innermost white circle represents the concepts supported by PAP: mental injuries diagnosed as a multidimensional spectra treated holistically.

Beyond potential novel treatments, PAP has important practical and theoretical consequences for the three axes of the crisis. The combination of psychotherapy with psychedelics can be conceptualized as the induction of an experience with positive long-term mental health consequences, rather than daily neurochemical corrections in brain dysfunctions (Figure 1). Thus, a comprehensive understanding of PAP suggests a conceptual expansion of “drug efficacy” to “experience efficacy^4^” Instead of conceiving the drug as correcting functional imbalances in the brain through a specific receptor, PAP is a treatment modality in which specific pharmacological actions temporally induce modifications in brain functioning and conscious experience. When appropriately mediated, these can be deeply meaningful experiences that elicit the emotional, cognitive and behavioral changes reported. Attempts to develop ketamine and ibogaine analogs devoid of the subjective “psychedelic” effects, e.g., lanicemine and 18-MC, will further illuminate this question. However, available therapeutic results for depression with ketamine analogs with less dissociative effects were only modest (Iadarola et al., 2015), while ketamine administration without preparatory psychotherapy and music support recently resulted in an interrupted trial (Gálvez et al., 2018). Furthermore, positive correlations between subjective features like ketamine's dissociative effects (Luckenbaugh et al., 2014) or psilocybin peak-experience with positive treatment outcomes in depression (Roseman et al., 2018) corroborates the notion that the meanings of the psychedelic experience plays an important role in therapeutic outcomes (Grof, 2008; Hartogsohn, 2018). It is thus very hard to strictly reduce PAP to neuropharmacology. In this sense, PAP can benefit from potentially rich interactions with other fields like psychodynamic psychotherapy (Plakun, 2006, 2012). Furthermore, PAP can help solve many pressing safety concerns in current psychopharmacological treatments by bridging a current gap in knowledge between research and clinical practice. This gap is created because psychiatric clinical trials rarely last longer than 6 months (Downing et al., 2014), while the products approved based on these trials are later prescribed for chronic daily use for years, sometimes decades. Many current adverse consequences from the use of psychiatric prescription medications arise from this gap, including decreasing drug adherence over time (Chapman and Horne, 2013; Medic et al., 2013), toxicity from increasing polypharmacy (Mojtabai and Olfson, 2010; Kukreja et al., 2013), addiction to prescribed medications causing severe withdrawal symptoms (Wright et al., 2014; McHugh et al., 2015; Novak et al., 2016), and a plethora of side effects arising after prolonged daily drug use, e.g., weight changes, stomach pains, constipation, mood swings, confusion, abnormal thoughts, delusions, memory loss, restlessness, akathisia, tardive dyskinesia, sexual dysfunction, anxiety, dizziness, sleep problems, and even suicidal ideas^5^ By administering medications only under supervision, PAP can reduce or even eliminate drug adherence problems and polypharmacy. By administering psychoactive drugs just a few times, PAP can prevent addiction and the development of side effects after chronic use of medications. And by exclusively licensing psychedelics for especially licensed therapists and physicians, rather than prescription and dispensation to patients, PAP can reduce risks of diversion and abuse. Considered together, these PAP features can arguably help reduce psychiatry's alarmingly high-rate of post-market safety events, reported at more than 60% after 10 years (Downing et al., 2017).

Besides critical consequences for the therapeutic axis, PAP is also relevant for diagnostic concerns. The fact that ketamine and psilocybin, substances with radically different pharmacological mechanisms of action, can induce positive outcomes in a single disorder, like depression; or that a single substance like psilocybin can be used to treat different disorders, like depression or drug dependence, challenges nosologies which discriminate disorders in mutually-exclusive categories. Thus, PAP supports a multidimensional spectra (Figure 1). However, proposals such as the RDoC were criticized by its biomedical reductionism (Frances, 2014a; Parnas, 2014; Wakefield, 2014), while psychedelic research recognize the concept of set and setting (Eisner, 1997; Hartogsohn, 2016) as crucial for the results obtained with these treatments. Set includes circumstances and factors other than drug and pharmacological targets, including people's beliefs, attitudes, preferences, choices and motivations. Setting refers to environment, context, therapists, supporting team etc. Thus, PAP supports other conceptually richer diagnostic approaches considerate of biopsychosocial factors (Frances, 2014b; Lefèvre et al., 2014; Borsboom, 2017; Johnstone, 2017).

This does not imply that neuroscience is not fundamental to understanding PAP and its consequences for psychiatric research and development. On the contrary. Current limitations of neuroimaging in psychiatry include long-term confounders like smoking, weight and metabolic variations (Linden, 2012; Weinberger and Radulescu, 2016), and low prognostic accuracy and predictive validity (Linden, 2012; Berkman and Falk, 2013; Weinberger and Radulescu, 2016). By developing faster treatments and bridging the gap between research and clinical practice, PAP can allow the use of within-subject designs in shorter time spans (e.g., Carhart-Harris et al., 2017b), reducing the impact of confounders and improving reliability of neuroimaging data. Thus, confidence in translating results from acute psychedelic neuroimaging (Vollenweider and Kometer, 2010; Muthukumaraswamy et al., 2013; Carhart-Harris et al., 2014a,b, 2017b; Tagliazucchi et al., 2014; Kraehenmann et al., 2015; Scheidegger et al., 2016; Lewis et al., 2017; Schartner et al., 2017) to clinical applications which will more closely resemble research designs is increased.

Finally, detailed study of the subjective aspects of PAP has enormous consequences for the explanatory axis. Recent qualitative and phenomenological research shows that psychedelic experiences involve meaningful autobiographical and social psychological concerns (Grof, 2008; MacLean et al., 2011; Turton et al., 2014; Baggott et al., 2015; Gasser et al., 2015; Schenberg et al., 2016, 2017; Belser et al., 2017; Liechti et al., 2017; Nour and Carhart-Harris, 2017; Watts et al., 2017). Therefore, PAP can deepen understanding of which psychological contents of the therapeutic experience are most relevant for treatment outcomes (Nour et al., 2016; Preller and Vollenweider, 2016; Schenberg et al., 2016, 2017; Carhart-Harris et al., 2017a). This can not only foster improvements in PAP but corroborates the importance of biopsychosocial aspects in psychiatric explanations. A rich methodological integration can help develop theoretical constructs that are not excessively reductionistic. Thus, PAP can conceptually enrich psychiatric explanations for mental disorders and their treatment. If neglect, trauma, childhood adversities, poverty, abuse, and deprivation—i.e., mental injuries—can have lasting negative consequences for mental health, it is also logically plausible that positive, cathartic experiences, sometimes of the mystical type, reliably achieved in PAP, can induce long lasting positive mental health outcomes. Indeed, in the 1950's and 60's, before drug scheduling and cessation of clinical studies with psychedelics, and before neuroscience took central stage in psychiatric understanding of mental disorders, pioneer psychiatrists like Stanislav Grof and Sidney Cohen already questioned the fundamental theoretical grounds of mental disorders (Grof, 1971, 1998, 2012; Cohen, 1972). Based on theirs' and others' experiences in non-ordinary states of consciousness with positive therapeutic outcomes (termed “holotropic” and “unsane,” respectively), they made radical theoretical proposals that can still be relevant to psychiatry, as it was for psychology (Grob and Bossis, 2017). It is thus possible that instead of brain dysfunctions causing discrete disorders treated with specific drugs, psychiatry can conceptualize mental injuries causing suffering that can be optimally treated with holistic approaches (Figure 1), including those which modulate the state of consciousness. This can greatly contribute to the understanding of how social circumstances and adverse life experiences shape mental health and brain activity, and how meaningful treatment experiences foster resilience.

## Author contributions

The author confirms being the sole contributor of this work and approved it for publication.

### Conflict of interest statement

As founder of a startup company the author has a potential conflict of interest due to his involvement with the design of clinical trials with psychedelics in Brazil. He has not been directly involved with any of the previous clinical trials cited in the article.

## Footnotes

^1^https://www.centerwatch.com/drug-information/fda-approved-drugs/therapeutic-area/17/psychiatry-psychology

^2^See also https://www.globaldrugsurvey.com/

^3^See MAPS treatment manual http://www.maps.org/research-archive/mdma/MDMA-Assisted-Psychotherapy-Treatment-Manual-Version7-19Aug15-FINAL.pdf

^4^https://www.youtube.com/watch?v=N64ynqSb9hk

^5^http://www.cchr.org/sites/default/files/The_Side_Effects_of_Common_Psychiatric_Drugs.pdf
